# Dendritic cells pulsed with placental gp96 promote tumor-reactive immune responses

**DOI:** 10.1371/journal.pone.0211490

**Published:** 2019-01-31

**Authors:** Huaguo Zheng, Lanlan Liu, Han Zhang, Fangming Kan, Shuo Wang, Yang Li, Huaqin Tian, Songdong Meng

**Affiliations:** 1 CAS Key Laboratory of Pathogenic Microbiology and Immunology, Institute of Microbiology, Chinese Academy of Sciences (CAS), Beijing, China; 2 Foshan Hospital of Traditional Chinese Medicine, Foshan, Guangdong, China; 3 Savaid Medical School, University of Chinese Academy of Sciences, Beijing, China; Duke University School of Medicine, UNITED STATES

## Abstract

Defining and loading of immunogenic and safe cancer antigens remain a major challenge for designing dendritic cell (DC)-based cancer vaccines. In this study, we defined a prototype strategy of using DC-based vaccines pulsed with placenta-derived heat shock protein gp96 to induces anti-tumor T cell responses. Placental gp96 was efficiently taken up by CD11c+ bone marrow-derived DCs (BMDCs) and resulted in moderate BMDC maturation. Splenocytes and cytotoxic T cells (CTLs) generated with mouse BMDCs pulsed with placental gp96 specifically lysed B16 melanoma and LLC lung carcinoma cells. In both transplantable melanoma and lung carcinoma mice models, immunization with placental gp96-stimulated BMDCs led to a significant decrease in tumor growth and mouse mortality with respect to mice treated with liver gp96-pulsed BMDCs or placental gp96 alone. This vaccine induced strong cross-reactive tumor-specific T cell responses. Our results revealed that DCs pulsed with placenta-derived gp96 represent an effective immunotherapy to induce tumor-reactive immune responses, possibly via loading DCs with its associated carcinoembryonic antigens.

## Introduction

As professional antigen-presenting cells (APCs), dendritic cells (DCs) can initiate naïve T cell responses and boosting memory T cell responses. The key function of DCs is to capture antigens and present antigenic peptides to naïve T cells to launch an adaptive immune response. In addition, they are able to recognize pathogen-associated molecular patterns (PAMPs) or damage-associated molecular patterns (DAMPs) and undergo maturation with increased expression of surface MHC molecules, costimulatory molecules, chemokine receptors, and secretion of cytokines (*e*.*g*., IL-12, IL-4, IL-6, TGF-β, and IL-10) [1, 2]. The unparalleled ability of DCs to prime CD4+ T helper cells and CD8+ cytotoxic T lymphocytes (CTLs), and orchestrate the adaptive immune response against neoplasm, has attracted increasing attention for the development of cancer vaccines [3].

A major challenge for designing effective therapeutic or prophylactic DC-based cancer vaccines is to define immunogenic cancer antigens that act as targets for immunological recognition. Such targets typically include tumor-specific antigens (*e*.*g*., mutated neoantigens, cancer germline proteins, and viral proteins) and tumor-associated antigens (TAAs) that are encoded by tissue-specific genes but over-expressed in tumor cells These antigens are loaded on DCs in the form of synthetic peptides, tumor-derived exosomes, autologous or allogeneic cell lysates from killed tumor cells or immunogenic cell death, or by transfecting DCs with tumor-derived whole RNA or DNA [4–7]. Presently, a limited repertoire of cancer antigens for DC loading has been identified to be immunogenically efficient that could elicit an immune response and result in tumor eradication, such as MAGE antigens, gp100, α-fetoprotein (AFP), glypican-3 (GPC-3), Wilms tumor 1, NY-ESO-1, MUC1, and mutated neoantigens [1, 4, 8]. In addition, DC adjuvants are also key to induce DC maturation and activation and, thus, intensify the immune response. Various combinations of cytokines, TLR agonists, and CD40 ligands have been utilized with different DC vaccines [4, 9].

Heat shock protein gp96 belongs to the HSP90 family. As an endoplasmic reticulum (ER)-resident chaperone, gp96 has the unique ability to associate with antigenic peptides of 5–25 amino acids in length. Cellular gp96, along with other HSPs including hsp70, hsp90, and calreticulin, constitutes a relay line for the efficient transport of endogenous peptides from the proteasome to MHC I in a cooperative manner [10]. Under subcutaneous immunization, gp96-peptide complexes traffic to draining lymph nodes and are predominantly taken up by CD11b+ and CD11c+ APCs [11]. The immunogenic gp96 complexed with antigenic peptides is internalized into DCs through its CD91 or scavenger receptor-A (SR-A) receptor, facilitating the antigen uptake by up to two orders of magnitude [12]. Additionally, the interaction of gp96 with CD91 or Toll-like receptors (TLRs) on APCs triggers downstream signaling cascades to activate nuclear factor-kappa B, leading to cell maturation and secretion of Th1-type cytokines [13, 14]. Immunization with DCs pulsed with tumor-derived gp96 induces effective CTL and Natural Killer (NK) responses and results in tumor inhibition in mice [15].

It is well documented that cancer and embryonic tissues have overlapping expression spectrums, and most solid tumors express embryonic antigens to some extent. As a temporary embryonic organ, the placenta expresses abundant TAAs, including IGF2, HIF-2a, GPC3, pregnancy-associated plasma protein A (PAPP-A), and MUC1 [16, 17]. Indeed, vaccination with embryonic or stem cell antigens leads to a potent protective immune response against cancers [18, 19]. Our previous study demonstrates that gp96 from placenta administrated as a prophylactic and therapeutic cancer vaccine exhibits anti-tumor T cell responses against melanoma and breast cancer in mice. Further analysis revealed that placental gp96 associates with peptides from carcinoembryonic antigens HER2 and MUC1 [20]. Unlike tumor tissues that are difficult to obtain, the placenta is readily available as it is generally discarded after birth. The anti-tumor efficacy of placental gp96-stimulated DCs was investigated in this study. The results may offer a new immunotherapeutic strategy against cancer by targeting carcinoembryonic antigens.

## Methods

### Mice and ethics statement

All animal experiments were carried out in strict accordance with institutional guidelines on handling of laboratory animals. The study design, monitory protocol and humane endpoint criteria were approved by the Research Ethics Committee of Institute of Microbiology, Chinese Academy of Sciences (approved number PZIMCAS2011001). Female C57BL/6 (6–8 weeks old) mice were purchased from the Animal Research Center of the Medical Department of Peking University (Beijing, China) and housed under specific pathogen-free (SPF) conditions in cages of 5 animals with water and food *ad libitum*. After tumor inoculation, animal health was monitored every other day for signs of distress or pain. Only one tumor was implanted in each mouse and mice were euthanized by cervical dislocation when tumors reached a maximal diameter of 2.0 cm. In addition, animals with observable difficulty in moving, lethargy or skin ulceration due to tumor growth were also regarded as reaching humane endpoint and euthanized even though maximal diameter was less than 2.0 cm.

### Cell lines

B16-F10 metastatic murine melanoma cell lines and mouse Lewis lung carcinoma (LLC) cells were obtained from the American Type Culture Collection (Rockville, MD, USA). B16-F10 and LLC cells were cultured in Dulbecco’s modified Eagle’s medium (DMEM) containing 10% heat-inactivated fetal bovine serum, 1 g/L of glucose, 1 mmol/L of glutamine, 100 U/mL of penicillin, and 100 μg/mL of streptomycin, and incubated in 5% CO_2_ at 37°C.

### Preparation of gp96

Gp96 protein from C57BL/6 mouse placenta or liver tissue was extracted as previously described [21] by ConA-Sepharose affinity and anion exchange chromatography. Concentration of endotoxin was lower than 1 EU/mg measured by the Limulus Amebocyte Lysate assay (BioWhittaker, Walkersville, MD) and gp96 was labeled with a FITC Protein Labeling Kit (Invitrogen, Carlsbad, CA).

### Preparation of BMDCs and BMDC vaccine

BMDCs were cultured according to the method provide by Inaba [22]. In Brief, C57BL/6 mouse bone marrow cells were cultured in the presence of 20 ng/mL recombinant murine GM-CSF (R&D Systems) for seven days to acquire BMDCs. On day 7, BMDCs were harvested and stimulated with 100 μg/mL placental gp96 or 1 mg/mL B16-F10 or LLC cell lysates, respectively, for 4 h at 37°C. Tumor lysates were obtained from cell culture through three times of repeated freezing and thawing in liquid nitrogen, followed by centrifugation to remove cell debris. As the concentration of potential tumor antigens in cell lysates may be much lower compared to that of tumor-associated antigens within gp96-peptide complexes, more amount of tumor cell lysates was used to pulse BMDCs. After washing twice BMDCs were resuspended in PBS for subsequent immunization.

### Maturation and gp96 binding analysis of BMDCs

For maturation analysis, seventh-day BMDCs were resuspended to 1×10^6^ cells/mL in RPMI 1640 supplemented with 10% FBS and inoculated with 100 μg/mL of placental gp96 or mouse serum albumin (MSA) for 4 h. After washing twice, BMDCs were cultured for another 24 h and then stained for fluorescent antibodies against CD11c, CD80, CD83, CD86, and MHC II (eBioscience, CA, USA). Or 1×10^6^ seventh-day BMDCs were incubated with 100 μg/mL of FITC-conjugated placental gp96 in a total volume of 100 μL at 37°C for 2 hours and fixed by paraformaldehyde at different time points for the binding assay. For fluorescence staining, BMDCs were incubated with 100 μg/mL of FITC-labelled gp96 for 30 min before being fixed by paraformaldehyde and stained by DAPI (Invitrogen, USA). The fluorescence intensity was analyzed using a FACS Calibur and FlowJo software (Tree Star Inc.).

### Mouse vaccination and monitoring of tumor models

For prophylaxis studies, each C57BL/6 mouse was immunized by subcutaneous immunization in the abdominal flank with 4–5×10^5^ placental gp96-, liver gp96-, or tumor lysate-pulsed BMDCs or 20 μg of placental gp96 alone at weeks 1, 2, and 4. Similar number of CD11c+ BMDCs was used for each mouse immunization. One week after the third immunization, each mouse was subcutaneously challenged with 5×10^4^ B16-F10 cells/mouse or 1×10^6^ LLC cells. In therapeutic studies, C57BL/6 mice were first subcutaneously challenged with B16-F10 or cells. Beginning from the second day, mice were treated with placental gp96-, liver gp96-, or tumor lysate-pulsed BMDCs or 20 μg of placental gp96 alone four times at 3 or 4 days interval. Each group contained five mice. Tumor size was measured every 2–3 days and volume was determined using the formula V = (a^2^b)/2, in which a and b represents the smallest diameter and the largest diameter. Mice were monitored for 50 days for survival.

### Enzyme-linked immunosorbent spot (ELISPOT) analysis

Mouse IFN-γ ELISPOT assays were performed according to the protocol supplied by manufacturer (Mabtech, Mariemont, OH). Briefly, isolated mouse splenocytes were added to the wells in triplicate with different stimuli and incubated at 37°C for 24 h. Spot forming cells (SFCs) were counted and analyzed with an ELISPOT Reader (Biosys, Germany).

### T cell cytotoxicity assay

Cytolysis was determined by 5-(6)-carboxy-fluorescein succinimidyl ester (CFSE) and propidium iodide (PI) staining. Mouse splenocytes were used as effector cells and B16-F10 or LLC cells as target cells. Target cells were pre-labeled with CFSE and mixed with effector cells at various ratios in a final volume of 200 μL for 5 h of incubation at 37°C. Dead cells were labeled with PI and samples were analyzed using FACS Calibur and FlowJo software (Tree Star Inc.).

### Statistical analysis

Reported values are expressed as means±s.d. Differences between groups were compared by two-tailed Student’s *t*-tests and differences between Kaplan-Meier curves were compared with the log-rank test. *P*-values <0.05 were considered significantly different. In figures * indicates for *P*<0.05, ** for *P*<0.01 and *** for *P*<0.001.

## Results

### Placental gp96 induces moderate DC maturation

**Fig 1:**
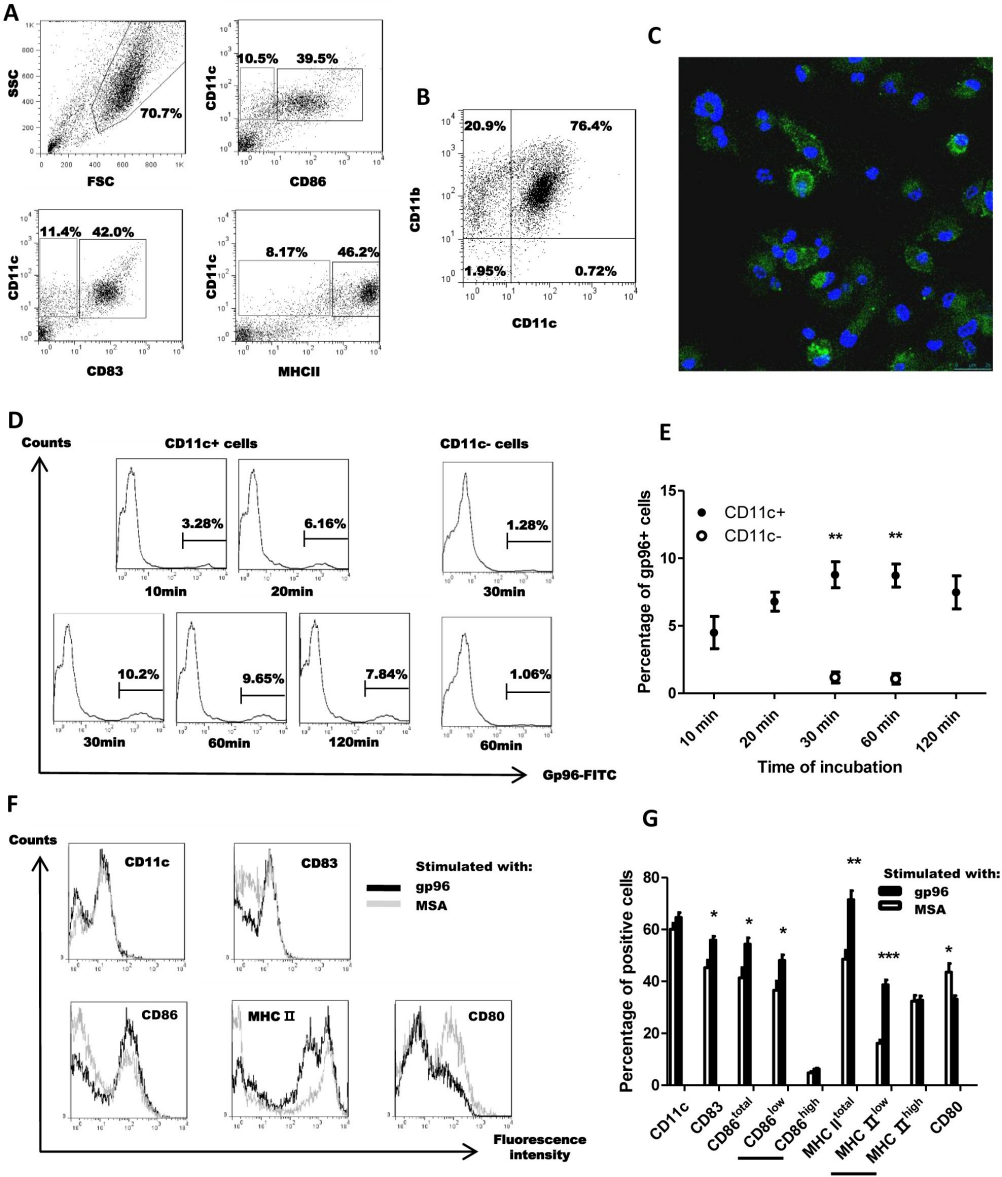
Placental gp96 binds with BMDCs and increases percentages of CD83^+^, CD86^low^ and MHC II^low^ populations. (A) BMDCs of the seventh day were phenotyped for surface markers. (B) Expression of CD11c and CD11b. (C) Fluorescence staining of BMDCs after incubation with FITC-labelled gp96 for 30 minutes. (D) After incubation with FITC-labeled gp96 for the indicated times, percentage of FITC positive BMDCs within CD11c+ and CD11c- subpopulation were analyzed by FACS. (E) Time curve of binding kinetics was shown. (F) After binding with gp96, phenotypical changes of BMDCs were analyzed. (G) Positive rates of each marker were shown. * indicates for *P*<0.05, ** for *P*<0.01 and *** for *P*<0.001. Data are representative of two independent experiments and FACS analyses were performed with three replicates in each experiment.

By the seventh day of culture, plenty of DC-like cells were observed detached from the bottom of culture dish and floating in the medium. The floating cells with typical DC morphology were collected and stained for BMDC markers CD11c, MHC II and costimulatory molecules CD83, CD86. Fig 1A shows a representative FACS result. Among the gated cells with relatively higher FSC and SSC, CD11c^+^CD83^+^, CD11c^+^CD86^+^, and CD11c^+^MHC II^+^ double positive cells accounted for 42%, 39.5%, and 46.2%, and the percentages of CD11c+/CD83-, CD11c+/CD86-, CD11c+/MHCII- cells were 11.4%, 10.5% and 8.17%, respectively. Most (76.4%) of the cultured BMDCs were CD11b and CD11c double positive (Fig 1B). As shown in Fig 1C (see S1 Fig for full image), the percentage of FITC positive BMDCs with variable fluorescence intensity was around 54% by immunofluorescence staining, which evidences that gp96 can be efficiently taken up by these BMDCs [11]. The percentage of FITC-gp96-positivity in CD11c+ cells by FACS analysis peaked (10.2%) at 30 min after incubation (Fig 1D). Compared with CD11c- cells, CD11c+ cells are much more efficient in taking up gp96 (Fig 1E, CD11c+ *vs*. CD11c-, *P*<0.01, at 30 min, see S1 Table for detailed results). We speculate that the discrepancy between fluorescence staining and FACS analysis may be due to the rapid internalization and degradation of FITC-labeled gp96 within BMDCs, resulting in the weakly fluorescent cells hardly detected by flow cytometer. It is also possible that the decreased binding is due to downregulation of gp96 receptor in activated BMDCs [23].

After stimulation by gp96, BMDCs exhibited significant phenotypical changes. Cell surface expression of CD83, CD86, and MHC II was significantly increased while CD80 was significantly decreased after gp96 stimulation (Fig 1F and 1G). The percentages of CD86^low^ and MHC II^low^ population were significantly increased but CD86^high^ and MHC II^high^ population were only slightly increased after stimulation (Fig 1G, see S1 Table for detailed results), indicating that gp96 induced moderate BMDC maturation.

### The prophylactic and therapeutic antitumor efficiency of a placental gp96-pulsed BMDC vaccine

**Fig 2:**
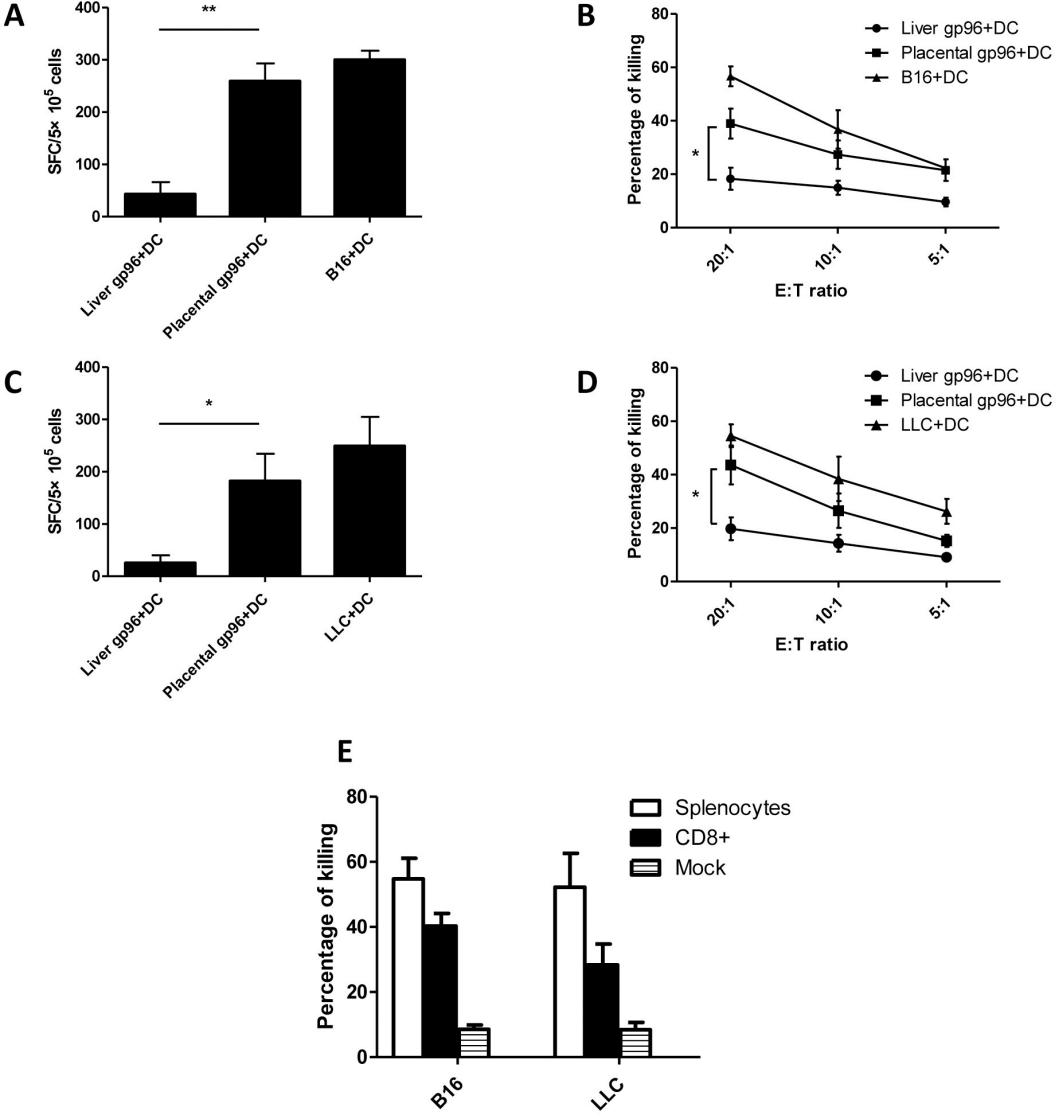
*Ex vivo* activation of antitumor CTLs by BMDCs pulsed with placental gp96. After incubation with BMDCs pulsed by different gp96 or tumor lysates, splenocytes from naïve mice were analyzed. (A) IFN- γ ELISPOT with B16-F10 lysate as stimulation. (B) Cytotoxicity against B16-F10. (C) IFN- γ ELISPOT with LLC lysate as stimulation. (D) Cytotoxicity against LLC. (E) Cytotoxicity of whole splenocytes and isolated CD8+against B16-F10 and LLC. * indicates for *P*<0.05, and ** for *P*<0.01. Each experiment was carried out with three replicates and the data are representative of two independent experiments with similar results.

At first, the immunogenicity of a placental gp96-pulsed DC vaccine was determined *in vitro*. Splenocytes from naïve C57BL/6 mice were co-cultured with BMDCs pulsed with mouse placental gp96, mouse liver gp96 as negative control, or B16 melanoma cell lysate as positive control for 2 weeks. IFN-γ ELISPOT assays using B16-F10 cell lysate for stimulation revealed that placental gp96-pulsed BMDCs could remarkably induce B16-F10-specific immune responses (Fig 2A). High cytotoxicity against B16-F10 target cells was also observed (Fig 2B). Immune responses and cytotoxicity against LLC lung carcinoma were observed after naïve splenocytes were co-cultured with gp96-stimulated BMDCs (Fig 2C and 2D). As shown in Fig 2E, the cytotoxicity of isolated CD8+ cells accounted for more than half of that of splenocytes, indicating that CTLs may play a major role in BMDC-induced antitumor immune responses. Detailed results are provided in S2 Table.

**Fig 3:**
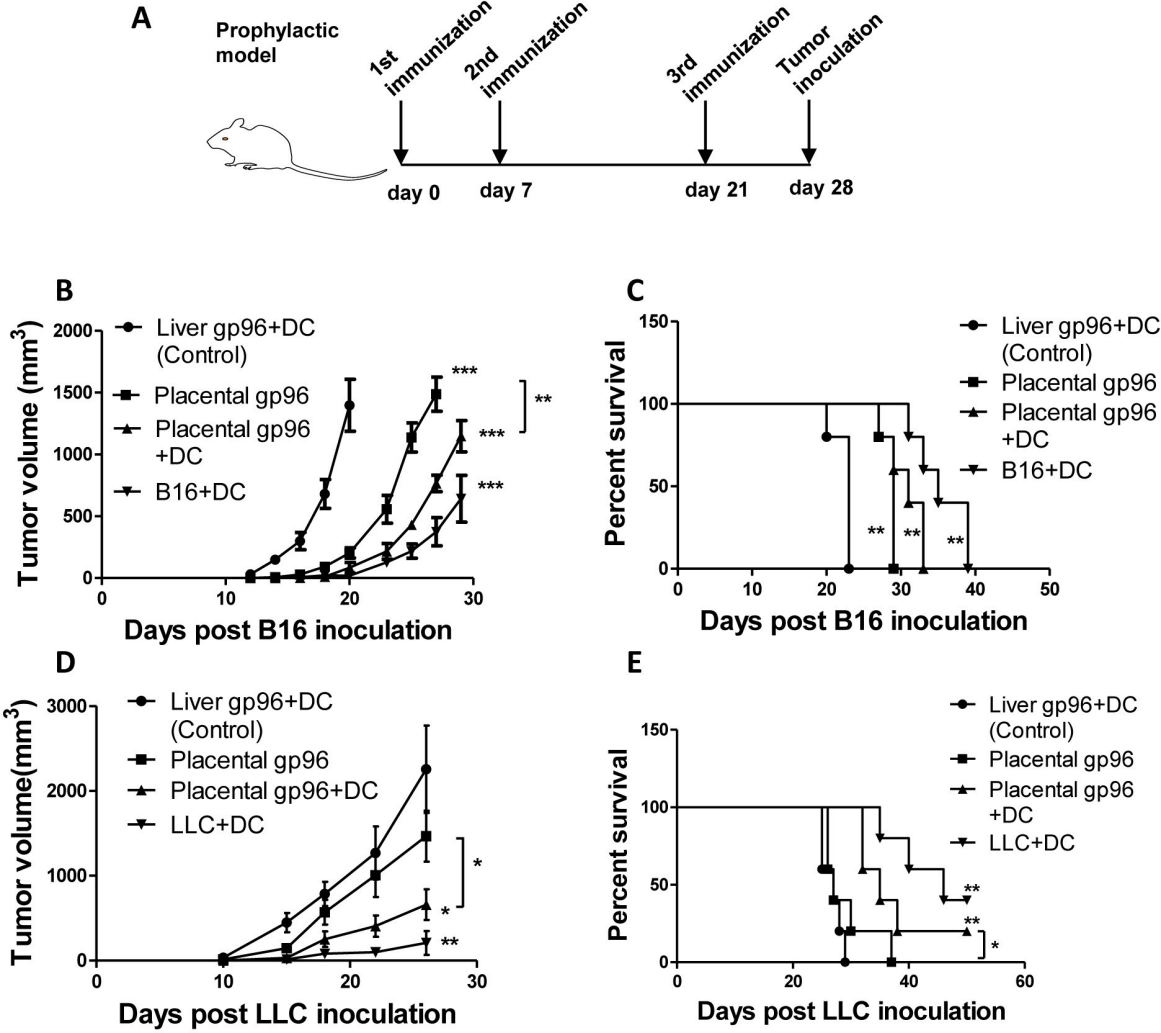
Immunization with placental gp96-pulsed BMDCs induces prophylactic effect against B16-F10 and LLC tumor models. For prophylactic analysis: (A) Representative diagram of vaccination strategy for prophylactic model. (B) Growth curve of B16-F10 tumor. (C) Kaplan-Meier survival curve of B16-F10 tumor-burdened mice. (D) Growth curve of LLC tumor. (E) Kaplan-Meier survival curve of LLC tumor-burdened mice. * indicates for *P*<0.05, ** for *P*<0.01 and *** for *P*<0.001. Data show mean ± SD of five mice. Data are representative of two independent experiments with similar results.

Next, we tested the ability of the placental gp96-pulsed DC vaccine to prevent tumors using a B16-F10 melanoma or Lewis lung cancer model. As shown in Fig 3A, after three times of immunization mice were subcutaneously challenged with B16-F10 or LLC cells and tumor size was measured. Compared with liver gp96-pulsed BMDCs, immunization with placental gp96-pulsed BMDCs significantly inhibited growth of both B16-F10 (Fig 3B, *P*<0.01) and LLC (Fig 3D, *P*<0.05) tumor model. In addition, an increased tumor suppressive effect was observed with placental gp96-stimulated BMDCs compared to placental gp96 immunization alone (*P*<0.01 for B16-F10 and *P*<0.05 for LLC model). Placental gp96-pulsed BMDC immunization also dramatically enhanced the survival time of tumor-burdened mice (Fig 3C and 3E, *P*<0.01 for both B16-F10 and LLC model), and prolonged mice survival compared with placental gp96 immunization alone (*P*<0.05 for LLC model). Detailed results are provided in S3 Table.

**Fig 4:**
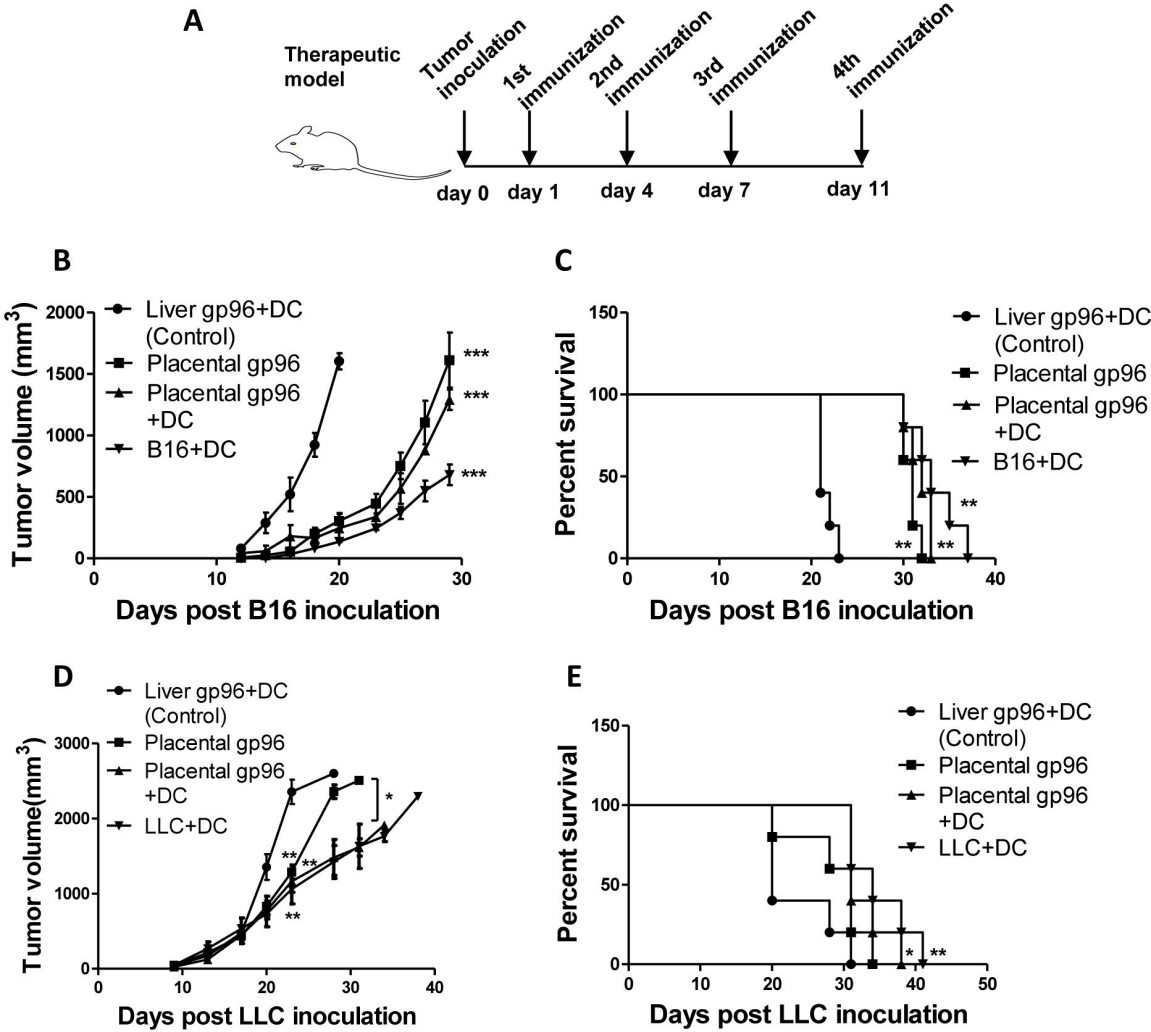
Immunization with placental gp96-pulsed BMDCs induces therapeutic effect against B16-F10 and LLC tumor models. (A) Representative diagram of vaccination strategy for therapeutic model. (B) Growth curve of B16-F10 tumor. (C) Kaplan-Meier survival curve of B16-F10 tumor-burdened mice. (D) Growth curve of LLC tumor. (E) Kaplan-Meier survival curve of LLC tumor-burdened mice. * indicates for *P*<0.05, ** for *P*<0.01 and *** for *P*<0.001. Data show mean ± SD of five mice. Data are representative of two independent experiments with similar results.

We also examined the therapeutic efficacy of BMDC vaccine in both tumor models. As shown in Fig 4A, following subcutaneous inoculation of B16-F10 or LLC cells, mice received injection of different BMDC vaccines or placental gp96 alone for four times. In accordance with the prophylactic models, treatment with placental gp96-pulsed BMDCs significantly inhibited B16-F10 (Fig 4B, *P*<0.001) and LLC (Fig 4D, *P*<0.01) tumor growth and improved mouse survival (Fig 4C and 4E, *P*<0.01 for B16-F10 and *P*<0.05 for LLC) compared with liver gp96-pulsed BMDCs. Placental gp96-pulsed BMDCs treatment also exhibited superior efficacy in both decreasing tumor volume and prolonging mice survival compared with gp96 immunization alone (Fig 4B–4E, *P*<0.05 in 4D). Detailed results are provided in S4 Table.

### Induction of antitumor T cell responses by placental gp96-stimulated BMDCs

**Fig 5:**
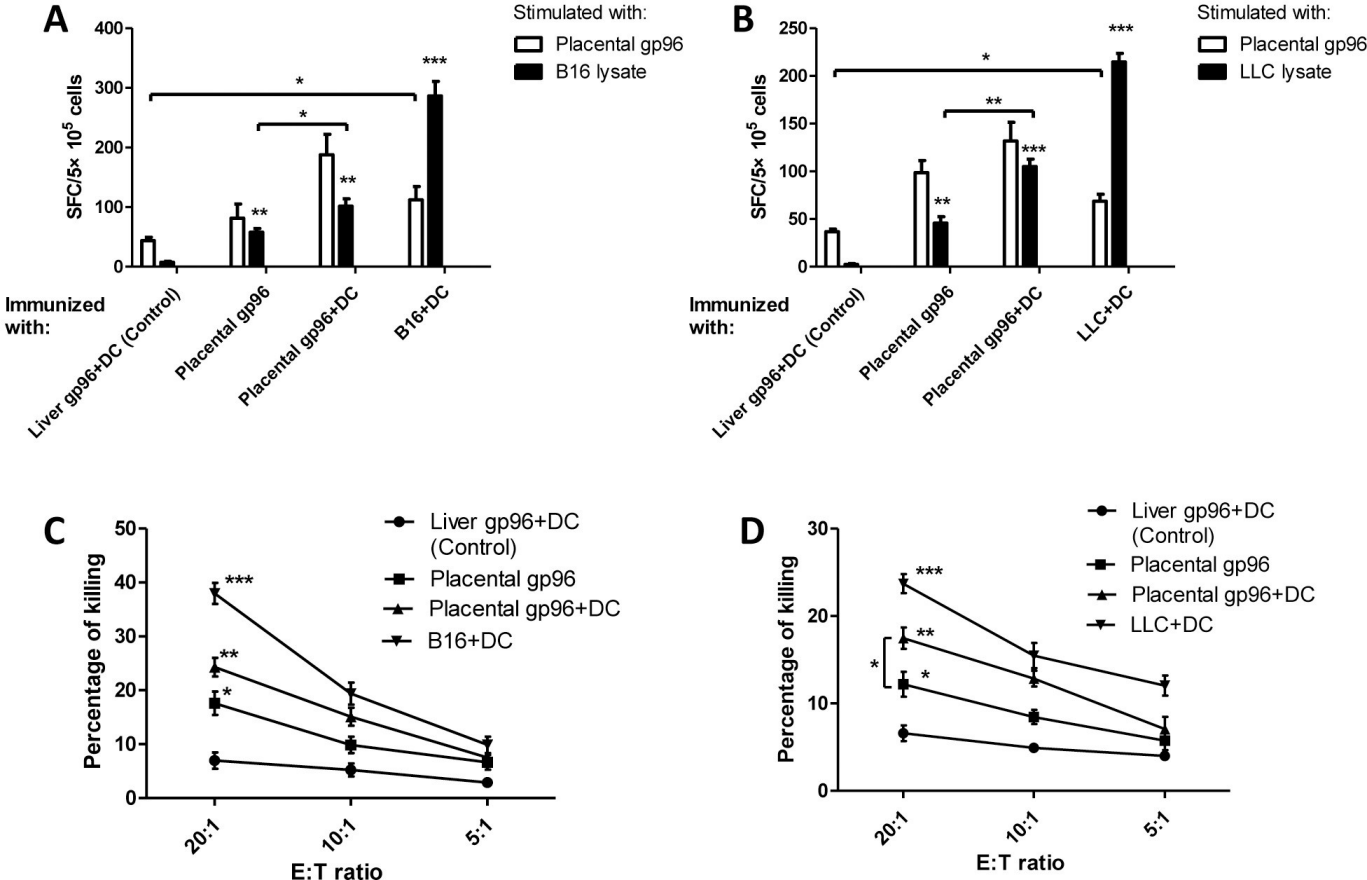
Immunization with placental gp96-pulsed BMDCs induces antitumor T cells specific for B16-F10 and LLC. Following immunization with differently pulsed BMDCs, splenocytes were analyzed. (A) IFN-γ ELISPOT with B16-F10 lysate or placental gp96 as stimulation. (B) IFN-γ ELISPOT with LLC lysate or placental gp96 as stimulation. (C) Cytotoxicity with B16-F10 as target cell. (D) Cytotoxicity with LLC as target cell. * indicates for *P*<0.05, ** for *P*<0.01 and *** for *P*<0.001. Data show mean ± SD of three replicates and are representative of two independent experiments with similar results.

Finally, we determined if the improved antitumor effect by placental gp96-stimulated BMDCs was due to induction of a specific T cell response. As shown in Fig 5A and 5B, an obvious T cell response against B16-F10 and LLC was observed in mice immunized with placental gp96-pulsed BMDCs (*P<*0.01 and 0.001, respectively, compared to liver gp96-pulsed BMDC immunization). Of note, splenocytes from mice immunized with B16 or LLC tumor lysate-pulsed BMDCs could also be activated under placental gp96 stimulation (both *P*<0.05 compared to liver gp96-pulsed BMDC immunization), suggesting antigen overlap between placental gp96-chaperoned peptides and tumor antigens from melanoma or lung carcinoma. In addition, splenocytes from placental gp96-pulsed BMDC-immunized mice also exhibited significantly higher cytotoxicity against B16-F10 and LLC target cells compared to those from mice immunized with liver gp96-pulsed BMDCs (Fig 5C and 5D). In concordance with previous results, placental gp96-pulsed BMDCs induced stronger tumor-specific T cell responses than placental gp96 immunization alone (Fig 5D, *P*<0.05). Detailed results are provided in S5 Table.

## Discussion

DC-based vaccines have emerged as a potent form of cancer immunotherapy. However, their antitumor efficiency is still limited, and the choice of the optimal antigen formulation still deserves extensive investigation. In this study, we developed a new DC-based vaccine for melanoma and lung carcinoma that combines the unique features of gp96 with highly immunogenic carcinoembryonic antigens from the placenta. We found that placental gp96, after internalization by mouse BMDCs, was able to induce moderate BMDC maturation and induced tumor-specific T-cells *in vitro*. Moreover, the placental gp96-pulsed DC vaccine (but not liver gp96-pulsed DC) possessed potent antitumor activity in both B16 melanoma and LLC lung cancer models. Further mechanistic study indicated that the placental gp96-based DC vaccine induced B16- and LLC-specific T cell response *in vivo*. Our current work demonstrates the feasibility and efficacy of placental gp96 as both a DC maturation stimulator and suitable tumor antigen for the development of antitumor DC-based vaccines.

As a natural adjuvant, gp96 has a unique immune-modulating activity in activation of innate immunity, as well as the capacity for antigenic peptide binding and antigen presenting. Autologous gp96 tumor vaccines isolated from resected tumors or gp96-Ig secreting vaccines from tumor cells have been tested for treatment of various cancers in clinical trials [24, 25]. However, the antitumor efficiency of gp96-based vaccines has been relatively modest, partly due counteracting immunosuppressive mechanisms in the tumor microenvironment. For example, some tumor cells constitutively express receptor-associated protein (RAP), a molecule that is able to abrogate the interaction between gp96 and CD91 [26]. In this study, BMDCs matured by placental gp96 were capable of inducing melanoma- or Lewis lung cancer-specific CTLs both *in vitro* and *in vivo*. Considering that placental gp96-pulsed BMDC immunization exhibited significantly stronger prophylactic and therapeutic efficacy compared to placental gp96 immunization alone (Figs 3 and 4), it’s plausible that the treatment of placental gp96 combined with BMDCs could bypass RAP-mediated CD91 blockage in the tumor microenvironment. Moreover, more investigation on the surface markers and secreted cytokines should be made to determine the relative percentage of conventional DC (cDC) and plasmacytoid DC (pDC) in the cultured BMDCs, as the latter subtype has been reported to have high ability in inducing regulatory T cells [27].

Unlike autologous tumor tissues with limited availability, the placenta is readily available as it is generally discarded after birth. Because highly specific tumor antigens are still lacking in most cancer types, pulsing DCs with whole tumor cell lysate represents a major direction in preparing DC-based tumor vaccines [28]. In this context, placental gp96 with overlapping carcinoembryonic antigens might be more attractive than tumor lysates and act as a multivalent vaccine aiming at precancerous cells undergoing dysplastic changes. Our study therefore provides the basis for the generation of placental gp96-baed DC vaccines on a large scale. In addition, given that higher antitumor efficiency was observed for tumor cell lysates-pulsed DC than placental gp96-pulsed DC, it is worthwhile to investigate the possibility of using gp96-TAA complexes reconstituted *in vitro*, or a combination of different tumor cell lysates as a universal therapeutic vaccine. Comprehensive analyses of the peptide repertoire associated with placental gp96 are needed to further dissect DC vaccine-mediated immune responses against different tumor types, which is under investigation in our laboratory.

In our study, major surface markers on matured DC such as CD86 and MHC II were moderately up-regulated after gp96 stimulation, except for CD80 which was down-regulated (see Fig 1E). However, the percentage increase in CD86^+^ and MHC II^+^ populations was mainly caused by increases in CD86^low^ and MHC II^low^ subpopulations. The moderate maturation efficacy of BMDCs by gp96 stimulation may be due to the relatively low expression of TLRs (e.g. TLR2/4/9) on BMDCs that may interact with gp96, as BMDCs induced by GM-CSF in our study were reported to be more immature before proper stimulation [29]. Given that CD80 could interact with both CD28 and CTLA-1 and thus plays an inhibitory role in T cell activation [30], gp96-induced down-regulation of CD80 may account for gp96’s superior ability to activate CTLs and initiate CD8-biased T cell responses [31]. As one of the most abundant proteins within cells, the abnormal increase of gp96, *e*.*g*., released from necrotic tumor cells, is an important immunological indicator that results in innate and adaptive immunity against undergoing neoplasm [32]. In our study, liver gp96-pulsed BMDC conceivably experience an identical maturation process but exhibited much less anti-tumor efficacy both *in vitro* and *in vivo*. This indicated that the adaptive anti-tumor immunity was mainly a result from cross-presentation of placental gp96-associated peptides rather than nonspecific immunopotentiation caused by matured BMDCs.

In summary, our study provides the proof-of-principle for utilizing gp96 from placenta as antigens and for stimulating DC maturation, laying the foundation for development of novel therapeutic DC-based cancer vaccines.

## Supporting information

S1 Table Detailed results for Fig 1. (DOCX)

S2 Table Detailed results for Fig 2. (DOCX)

S3 Table Detailed results for Fig 3. (DOCX)

S4 Table Detailed results for Fig 4. (DOCX)

S5 Table Detailed results for Fig 5. (DOCX)

S1 Fig Full image for fluorescence staining in Fig 1C. (TIF)
