# Correction: Dendritic cells pulsed with placental gp96 promote tumor-reactive immune responses

**DOI:** 10.1371/journal.pone.0218362

**Published:** 2019-06-10

**Authors:** Huaguo Zheng, Lanlan Liu, Han Zhang, Fangming Kan, Shuo Wang, Yang Li, Huaqin Tian, Songdong Meng

An additional affiliation for the first author is not indicated. Dr. Huaguo Zheng is also affiliated with: University of Chinese Academy of Sciences, Beijing, China

## References

[1] Zheng H, Liu L, Zhang H, Kan F, Wang S, Li Y, et al (2019) Dendritic cells pulsed with placental gp96 promote tumor-reactive immune responses. PLoS ONE 14(1): e0211490 10.1371/journal.pone.0211490 30703157 PMC6354997

