# Supplementary material for: Dendritic cells pulsed with placental gp96 promote tumor-reactive immune responses
Source: PLoS One. 2019 Jan 31;14(1):e0211490. doi: 10.1371/journal.pone.0211490 (PMC6354997; doi:10.1371/journal.pone.0211490)
Supplement: S1 Table — (DOCX) [file pone.0211490.s001.docx]

**Fig 1E Time curve of binding kinetics**

|  | Time points | CD11c+ population | | | CD11c- population | | |
| --- | --- | --- | --- | --- | --- | --- | --- |
| Percentage of FITC positive BMDCs (%) | 10min | 3.28 | 6.88 | 3.33 | -- | -- | -- |
|  | 20min | 6.16 | 8.20 | 6.01 | -- | -- | -- |
|  | 30min | 10.20 | 9.20 | 6.95 | 1.28 | 1.83 | 0.42 |
|  | 60min | 9.65 | 9.52 | 7.01 | 1.06 | 1.76 | 0.40 |
|  | 120min | 7.84 | 9.41 | 5.21 | -- | -- | -- |

“--” indicates for data not collected

**Fig 1G Positive rates change of markers after gp96 stimulation**

| (%) | Stimulated with MSA | | | Stimulated with gp96 | | |
| --- | --- | --- | --- | --- | --- | --- |
| CD11c | 63.30 | 61.30 | 55.80 | 62.40 | 68.4 | 63.10 |
| CD83 | 40.00 | 46.30 | 49.70 | 53.70 | 58.7 | 55.20 |
| CD86^total^ | 34.45 | 41.50 | 48.10 | 58.68 | 50.4 | 53.97 |
| CD86^low^ | 30.50 | 36.73 | 42.57 | 51.80 | 44.5 | 48.01 |
| CD86^high^ | 3.95 | 4.77 | 5.53 | 6.88 | 5.9 | 5.96 |
| MHC II^total^ | 46.80 | 55.20 | 43.67 | 71.70 | 65.3 | 77.40 |
| MHC II^low^ | 15.60 | 18.40 | 14.57 | 38.80 | 35.3 | 41.90 |
| MHC II^high^ | 31.20 | 36.80 | 29.10 | 32.90 | 30.0 | 35.50 |
| CD80 | 39.20 | 41.30 | 50.10 | 30.60 | 33.8 | 35.10 |
