# Supplementary material for: Dendritic cells pulsed with placental gp96 promote tumor-reactive immune responses
Source: PLoS One. 2019 Jan 31;14(1):e0211490. doi: 10.1371/journal.pone.0211490 (PMC6354997; doi:10.1371/journal.pone.0211490)
Supplement: S2 Table — (DOCX) [file pone.0211490.s002.docx]

**Figs 2A and 2C IFN-γ ELISPOT with B16-F10 and LLC as stimulant respectively**

|  | Liver gp96 + DC | Placental gp96 + DC | B16 + DC | Liver gp96 + DC | Placental gp96 + DC | LLC + DC |
| --- | --- | --- | --- | --- | --- | --- |
|  | B16-F10 as stimulant | | | LLC as stimulant | | |
| Spot counts per 5x10^5^ splenocytes | 87  10  33 | 255  202  320 | 311  323  266 | 52  2  23 | 101  280  166 | 271  143  333 |

**Figs 2B and 2D Cytotoxicity against B16-F10 and LLC cells**

|  | | Liver gp96 + DC | Placental gp96 + DC | B16 + DC | Liver gp96 + DC | Placental gp96 + DC | LLC + DC |
| --- | --- | --- | --- | --- | --- | --- | --- |
|  | E:T | B16-F10 as target cell | | | LLC as target cell | | |
| Cytotoxicity (%) | 20:1 | 21.0  23.7  10.2 | 39.0  48.6  29.3 | 51.2  55.1  63.7 | 28.3  16.1  14.9 | 55.1  45.7  30.1 | 62.1  54.3  47.3 |
|  | 10:1 | 10.2  15.3  19.3 | 30.1  34.8  17.1 | 38.7  48.2  23.4 | 20.1  9.2  13.7 | 38.6  24.3  16.7 | 45.4  48.1  21.8 |
|  | 5:1 | 9.1  12.6  7.1 | 22.8  27.8  14.0 | 24.7  22.8  19.3 | 12.3  6.4  8.6 | 15.4  19.1  11.2 | 35.1  24.3  19.4 |

**Fig 2E Cytotoxicity of whole splenocytes and isolated CD8+against B16-F10 and LLC.**

|  | Splenocytes | CD8^+^ cells | Mock | Splenocytes | CD8^+^ cells | Mock |
| --- | --- | --- | --- | --- | --- | --- |
|  | B16-F10 as target | | | LLC as target | | |
| Cytotoxicity (%) at E:T ratio of 20:1 | 58.8  63.1  42.3 | 40.6  46.8  33.5 | 8.24  11.02  6.53 | 42.3  73.0  41.5 | 16.2  37.9  31.0 | 5.17  7.3  12.8 |
