# Supplementary material for: Dendritic cells pulsed with placental gp96 promote tumor-reactive immune responses
Source: PLoS One. 2019 Jan 31;14(1):e0211490. doi: 10.1371/journal.pone.0211490 (PMC6354997; doi:10.1371/journal.pone.0211490)
Supplement: S3 Table — (DOCX) [file pone.0211490.s003.docx]

**Fig 3B Growth curves of B16-F10 tumor in prophylactic model.**

|  | Days post tumor inoculation | Liver gp96 + DC | Placental gp96 | Placental gp96 + DC | B16 + DC |
| --- | --- | --- | --- | --- | --- |
| **Tumor volume (mm^3^)** | **12** | 20.2  47.2  12.2  3.60  72.0 | 0.0  1.2  0.0  0.0  0.0 | 0.0  0.0  0.0  0.0  0.0 | 0.0  1.3  0.0  0.0  0.0 |
|  | **14** | 158.8  156.8  84.3  232.4  115.2 | 4.1  17.9  1.6  7.9  7.7 | 0.0  0.0  0.0  0.0  0.0 | 0.0  14.9  0.0  0.0  0.0 |
|  | **16** | 223.0  559.0  156.7  313.6  242.8 | 12.2  76.1  16.3  31.6  12.50 | 0.0  0.0  0.0  0.0  8.9 | 1.7  32.1  0.0  0.0  0.0 |
|  | **18** | 522.8  740.1  365.1  1055.0  720.3 | 39.6  81.0  106.8  179.6  54.8 | 5.8  0.0  0.0  4.6  45.7 | 3.9  54.6  0.0  1.2  3.3 |
|  | **20** | 1078.1  1871.2  1009.5  1630.9  END | 53.2  305.9  216.0  230.3  211.3 | 86.3  12.2  30.4  38.0  245.0 | 21.4  108.0  7.8  6.4  9.8 |
|  | **23** | END  END  END  END  -- | 261.1  884.3  739.6  423.7  475.3 | 198.5  111.4  111.6  198.6  459.3 | 140.4  267.2  71.4  86.3  68.5 |
|  | **25** | --  --  --  --  -- | 841.0  1368.0  1394.3  874.0  1211.0 | 529.9  443.1  312.7  420.3  445.0 | 348.8  361.3  169.0  86.3  125.0 |
|  | **27** | --  --  --  --  -- | 1498.0  END  1685.8  1091.5  1673.6 | 598.2  792.3  921.6  750.0  END | 725.1  541.5  250.0  81.3  275.1 |
|  | **29** | --  --  --  --  -- | END  --  END  END  END | 1183.0  END  1346.7  913.6  -- | 1310.4  786.5  465.8  247.4  400.0 |

“END” indicates for mouse dead during observation or euthanized for reaching humane endpoint.

“--” indicates for data unavailable as mice reached endpoint.

**Fig 3D Growth curves of LLC tumor in prophylactic model.**

|  | Days post tumor inoculation | Liver gp96 + DC | Placental gp96 | Placental gp96 + DC | LLC + DC |
| --- | --- | --- | --- | --- | --- |
| **Tumor volume (mm^3^)** | **10** | 17.6  18.0  30.9  22.5  80.3 | 6.4  38.4  20.5  3.2  3.4 | 0.0  0.0  1.0  0.0  1.2 | 0.0  0.0  0.0  0.0  0.5 |
|  | **15** | 380.7  352.0  396.1  225.4  885.6 | 120.6  324.8  171.0  80.3  37.4 | 10.1  0.0  55.5  27.9  62.5 | 29.8  38.8  0.0  4.0  0.0 |
|  | **18** | 1041.9  1183.0  505.8  728.3  469.6 | 384.8  936.0  907.5  384.0  235.2 | 99.6  0.0  257.1  445.5  455.5 | 120.0  277.7  0.0  5.4  0.0 |
|  | **22** | 1519.0  1544.5  426.5  2150.2  700.0 | 1058.0  981.6  1892.3  780.3  318.5 | 269.5  0.0  564.1  716.6  486.0 | 264.6  214.2  0.0  14.9  0.0 |
|  | **26** | 2629.9  2899.2  END  END  1239.1 | 1911.0  END  END  1594.3  893.0 | 541.9  0.0  958.0  939.0  859.1 | 725.1  302.5  0.0  14.0  0.0 |

“END” indicates for mouse dead during observation or euthanized for reaching humane endpoint.

“--” indicates for data unavailable as mice reached endpoint.
