# Supplementary material for: Dendritic cells pulsed with placental gp96 promote tumor-reactive immune responses
Source: PLoS One. 2019 Jan 31;14(1):e0211490. doi: 10.1371/journal.pone.0211490 (PMC6354997; doi:10.1371/journal.pone.0211490)
Supplement: S4 Table — (DOCX) [file pone.0211490.s004.docx]

**Fig 4B Growth curves of B16-F10 tumor in therapeutic model.**

|  | Days post tumor inoculation | Liver gp96 + DC | Placental gp96 | Placental gp96 + DC | B16 + DC |
| --- | --- | --- | --- | --- | --- |
| **Tumor volume (mm^3^)** | **12** | 90.0  24.0  136.1  3.0  152.1 | 9.2  0.0  0.0  6.4  15.3 | 101.4  101.3  0.0  0.0  6.2 | 0.0  3.4  0.0  0.0  0.0 |
|  | **14** | 281.8  168.2  337.5  86.4  572.2 | 27.6  6.0  8.6  34.4  55.2 | 30.6  238.1  0.0  2.5  27.0 | 0.0  4.5  7.9  0.0  4.0 |
|  | **16** | 416.8  451.6  1051.4  258.0  427.7 | 67.6  14.0  27.0  60.7  108.2 | 416.0  384.0  0.0  29.7  71.4 | 8.0  64.0  44.1  37.0  21.6 |
|  | **18** | 1081.9  688.5  1209.6  780.3  859.1 | 274.6  119.5  166.5  106.3  336.2 | 101.4  491.3  45.6  66.3  126.3 | 50.8  154.8  53.7  73.8  81.1 |
|  | **20** | 1655.28  1813.4  1622.0  1484.4  1441.6 | 410.4  196.5  175.8  225.4  509.9 | 174.1  701.1  81.3  110.4  166.5 | 140.4  156.3  87.1  128.6  166.5 |
|  | **23** | END  END  END  END  END | 558.6  363.9  238.1  375.5  689.1 | 317.7  760.0  198.5  198.0  215.3 | 203.4  360.2  155.6  278.4  221.4 |
|  | **25** | --  --  --  --  -- | 913.0  576.9  612.9  564.1  1098.5 | 416.0  989.6  712.9  346.8  374.5 | 323.2  544.6  299.6  406.8  272.5 |
|  | **27** | --  --  --  --  -- | 1242.8  724.6  968  865.2  1728.0 | 907.1  954.9  850.2  941.6  745 | 458.1  870.0  433.4  562.2  418.8 |
|  | **29** | --  --  --  --  -- | 2092.0  1122.8  1490.0  1178.6  2183.3 | 1451.6  1343.6  1326.7  1360.8  968.0 | 617.9  997.2  546.0  699.4  538.0 |

“END” indicates for mouse dead during observation or euthanized for reaching humane endpoint.

“--” indicates for data unavailable as mice reached endpoint.

**Fig 4D Growth curves of LLC tumor in therapeutic model.**

|  | Days post tumor inoculation | Liver gp96 + DC | Placental gp96 | Placental gp96 + DC | LLC + DC |
| --- | --- | --- | --- | --- | --- |
| **Tumor volume (mm^3^)** | **9** | 46.9  25.2  80.6  22.5  26.4 | 55.1  49.6  45.6  27.0  27.0 | 44.0  6.6  12.1  50.4  14.5 | 16.2  27.9  78.8  54.5  63.0 |
|  | **13** | 289.0  144.0  190.3  138.9  104.3 | 176.4  140.5  219.5  307.2  230.3 | 255.6  39.5  78.8  142.2  101.4 | 222.2  94.6  614.7  259.2  151.3 |
|  | **17** | 425.3  642.0  779.2  283.0  245.8 | 355.2  365.1  414.2  831.7  228.9 | 545.0  546.8  431.7  339.6  375.7 | 440.9  190.4  1047.9  418.2  566.5 |
|  | **20** | END  1525.9  1183.6  END  END | 732.1  517.6  1154.1  919.1  END | 844.0  799.3  712.9  417.2  1098.1 | 496.9  250.3  1245.5  901.5  754.9 |
|  | **23** | --  2518.3  2193.8  --  -- | 1015.6  1366.9  1421.0  1352.0  -- | 1391.7  699.4  1491.1  982.3  1250.0 | 1109.4  368.5  1364.3  953.3  1512.3 |
|  | **28** | --  2600.0  END  --  -- | 2436.6  END  2176.0  2462.5  -- | 2048.0  835.0  1856.3  1008.0  1664.0 | 1321.3  624.2  1800.0  1568.0  1804.4 |
|  | **31** | --  END  --  --  -- | 2508.8  --  END  END  -- | END  1731.4  END  1501.5  END | 1610.4  1132.6  END  2150.2  END |
|  | **34** | --  --  --  --  -- | END  --  --  --  -- | --  END  --  1912.5  -- | 1835.5  1692.5  --  END  -- |
|  | **38** | --  --  --  --  -- | --  --  --  --  -- | --  --  --  --  -- | 2296.4  END  --  --  -- |

“END” indicates for mouse dead during observation or euthanized for reaching humane endpoint..

“--” indicates for data unavailable as mice reached endpoint.
