# Supplementary material for: Dendritic cells pulsed with placental gp96 promote tumor-reactive immune responses
Source: PLoS One. 2019 Jan 31;14(1):e0211490. doi: 10.1371/journal.pone.0211490 (PMC6354997; doi:10.1371/journal.pone.0211490)
Supplement: S5 Table — (DOCX) [file pone.0211490.s005.docx]

**Fig 5A IFN-γ ELISPOT with B16-F10 lysates or placental gp96 as stimulant**

|  | Liver gp96 + DC | | Placental gp96 | | Placental gp96 + DC | | B16 + DC | |
| --- | --- | --- | --- | --- | --- | --- | --- | --- |
| **Stimulant** | P-gp96 | B16 | P-gp96 | B16 | P-gp96 | B16 | P-gp96 | B16 |
| **Spot counts per 5x10^5^ splenocytes** | 45  53  33 | 6  5  11 | 35  96  113 | 48  55  70 | 124  243  196 | 96  125  83 | 107  76  153 | 251  333  276 |

**Fig 5B IFN-γ ELISPOT with LLC lysates or placental gp96 as stimulant**

|  | Liver gp96 + DC | | Placental gp96 | | Placental gp96 + DC | | LLC + DC | |
| --- | --- | --- | --- | --- | --- | --- | --- | --- |
| **Stimulant** | P-gp96 | LLC | P-gp96 | LLC | P-gp96 | LLC | P-gp96 | LLC |
| **Spot counts per 5x10^5^ splenocytes** | 33  35  42 | 2  4  1 | 98  121  77 | 56  33  48 | 127  168  100 | 109  90  116 | 56  69  81 | 202  232  210 |

**Fig 5C Cytotoxicity with B16-F10 as target cell**

|  | E:T | Liver gp96 + DC | Placental gp96 | Placental gp96 + DC | B16 + DC |
| --- | --- | --- | --- | --- | --- |
| **Cytotoxicity (%)** | 20:1 | 9.6  4.3  7.0 | 17.0  21.6  14.2 | 21.8  27.6  23.5 | 34.2  41.0  38.8 |
|  | 10:1 | 7.2  3.0  5.4 | 10.3  12.3  7.0 | 14.2  18.3  12.8 | 15.6  22.7  19.8 |
|  | 5:1 | 3.4  2.1  3.2 | 6.5  9.1  4.3 | 7.4  8.9  6.4 | 7.1  12.3  10.3 |

**Fig 5D Cytotoxicity with LLC as target cell**

|  | E:T | Liver gp96 + DC | Placental gp96 | Placental gp96 + DC | LLC + DC |
| --- | --- | --- | --- | --- | --- |
| **Cytotoxicity (%)** | 20:1 | 8.3  5.3  6.1 | 9.37  13.2  14.0 | 16.9  15.7  19.8 | 22.0  25.7  23.4 |
|  | 10:1 | 5.4  4.0  5.3 | 6.82  9.4  9.1 | 13.0  11.2  14.3 | 12.7  16.2  17.5 |
|  | 5:1 | 4.5  3.2  4.2 | 3.68  6.2  7.3 | 6.1  5.3  9.8 | 10.6  14.3  11.2 |
