# Supplementary figures and images for: Dendritic cells pulsed with placental gp96 promote tumor-reactive immune responses
Source: PLoS One. 2019 Jan 31;14(1):e0211490. doi: 10.1371/journal.pone.0211490 (PMC6354997; doi:10.1371/journal.pone.0211490)

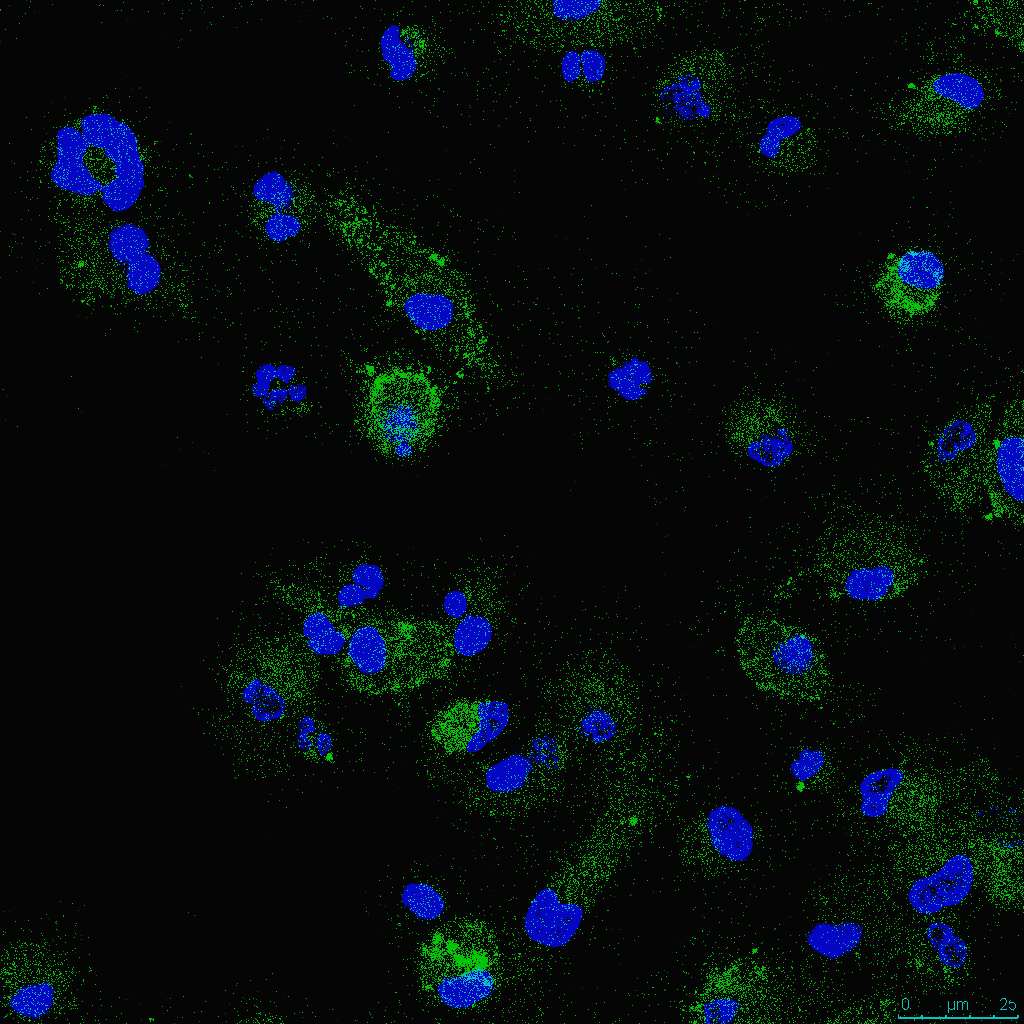

Supplement: S1 Fig — (TIF) [file pone.0211490.s006.tif]
